# Reports on Hospitals of the United Kingdom

**Published:** 1914-06-20

**Authors:** Henry Burdett


### REPORTS ON
### Hospitals of the United Kingdom.

By SIR HENRY BURDETT, K.C.B., K.C.V.O.

SERIES III.

### THE EPSOM AND EWELL COTTAGE HOSPITAL.

This institution was established in 1872. The new hospital, which was erected in 1889, has been built without much regard to cost, the plan being extravagant in the sense that it covers a great deal of ground and that much space is occupied by the portions which may properly be denominated administration buildings.

It is well equipped. It has an ample casualty department and operation theatre, and one of the most attractive private wards we have ever seen in a hospital of the kind—a painted ward, as it is called—which is somewhat elaborately decorated with paintings on the walls, with a male and female ward in addition. The lavatory and other adjuncts are entirely separate from the wards proper, and have to be approached by entering the main corridor of the hospital and proceeding to either end of it, where they are placed. The hospital contains sixteen beds, the average number occupied being rather more than half that number. Admission is by subscriber's letter or by payment of from 5s. to 15s. per week in the ordinary wards and three guineas per week in the private ward.

### A Type that is likely to Disappear.

The medical staff consists of nine medical officers and two dentists, but the serious cases are either sent to St. Thomas's Hospital or are treated by a surgeon obtained from London. This hospital is capable of doing most excellent work, and, so far as accommodation and appliances are concerned, there is no reason why it should not treat with success every class of case within its wards. As matters stand the amount of work actually done is small compared with what it might be, having regard to the size of the district and its requirements which this cottage hospital was established to meet. We gather, however, that it is the present policy of those who control these affairs to keep down the number of cases admitted and to restrict them as far as possible to the simplest type and accidents. It does not therefore fully meet, under the present policy, the requirements of the people throughout the district, a large number of whom, it is stated, seek hospital aid from some London hospital, which might properly be rendered by the Epsom and Ewell institution.

Free medical service is not apparently popular with the profession in this district. Recently a nursing home has been started a short distance away from the hospital which, it is thought, absorbs, and will continue to absorb, the patients who previously to this nursing home sought treatment at the cottage hospital. It follows that in the changes which are rapidly approaching, unless a different policy is pursued and preparations are made in advance, this institution may be regarded as one of a type which is likely to disappear.

It is interesting to note that, whereas the cottage hospital at Reigate has developed into a general hospital with forty beds, and does in the most competent manner all the medical and surgical work the needs of the inhabitants require, this cottage hospital at Epsom is credited with an opposite policy, with the possible result that the former will go on prospering and increase in interest and importance, while the latter under its present policy may in a few years simmer down to smaller and smaller proportions, so far as the work done is concerned, until it ultimately may cease to exist as a hospital.

### Medical Men and the Epsom Policy.

If the Epsom policy were to become popular with the younger members of the medical profession, quite one-half the existing cottage hospitals would probably be closed within the next fifteen or twenty years. We do not understand how any member of the medical profession, who is keen on his work, can regard such a policy as worthy of the best history of medicine, or calculated to have any but a damaging effect upon his own standard of work, seeing that it must ultimately deprive him of the means of keeping his hand in and helping him to secure material aid and facilities for the best work in his private practice. Having regard to changes in the population, and its large increase owing to local circumstances we need not here dwell upon, it seems to us that the sooner the education and county authorities look the matter in the face and provide a school clinic and other facilities for the treatment of the children in this district, the better, for they will thereby reflect credit upon themselves and follow the best traditions of up-to-date local government.

### The Way Out of the Clinic Problem.

Why should not the County Council purchase the Epsom and Ewell Cottage Hospital and convert it into a clinic of the best and most modern type? An alternative would be for the local authorities to make an arrangement with the hospital authorities to provide for the cases, which would otherwise go to such a clinic, on a *pro rata* basis—that is to say, an agreement would be entered into between these authorities to pay to the hospital the *pro rata* or actual cost of treating these cases at the hospital, plus 10 per cent. on the total cost of such treatment. This 10 per cent. would serve as the remuneration fund for the medical staff for services rendered and for others who shared in the work dealing with these *pro rata* cases.

### FAVERSHAM COTTAGE HOSPITAL.

This hospital was founded in 1888. It is built, and has its garden, in a gravel pit or brick earth, so that a large portion of the building is below the surface level of the street. Other buildings have been erected in Faversham on similar sites which are dry and well-lighted as this is.

The hospital contains eight beds and cots, in four wards, the larger ones having three beds each. Kitchens, dining room, and accommodation for the staff are in the basement. It has a well-proportioned operation theatre, which also contains an $x$-ray apparatus. This theatre was not at all well-kept, being in fact the least clean of all the operation theatres we have ever seen. An unusually large number of adenoid cases appear to be treated in this hospital.

There is a mortuary which contains a cupboard, two shelves, a *post-mortem* table, and other things. Adjacent to it is a chamber which, though practically under the same roof, has no direct connection with the mortuary. It was, we think, evidently intended to be a chapel or view room in connection with the mortuary, and in any case ought to be put in order and devoted to this purpose. At present it is kept locked, and here an ambulance of the St. John Ambulance Service is housed. Some other place should be found to store this ambulance, in order that the Faversham Cottage Hospital may adequately fulfil its duty by providing accommodation which will exhibit reverence for the dead and due regard for the feelings of the patients' friends.

There is a large garden which supplies the hospital with all its vegetables, except potatoes, throughout the year. We were sorry to notice a singular absence of flowers. In addition to a Work Guild—or as a portion of it—we shall hope to see a Ladies' Association formed to supply the hospital with linen and to organise a regular visitation of the hospital with a view to its being kept thoroughly smart. We formed the conclusion that a greater personal interest might be taken in the patients by the people of Faversham, as is the case nowadays almost everywhere throughout the country. The nursing staff, we gathered, consisted of a trained nurse and two probationers, in addition to the matron, so that the staff should be ample to make, and keep, this hospital beautifully clean and thoroughly smart throughout.

There are no soil sinks at present in the hospital, and some of the existing fittings might be improved. The hospital has ample funds, and this sanitary work, together with the provision of a footstool or platform to the bath and other matters, should be undertaken without delay. The bath referred to is raised some distance from the floor in order, it was explained, that the space around it might be readily swept. It is practically impossible for anyone easily to get into or out of this bath, as it is. In the case of the elder patients the bath is not usable with safety, and we recommend the improvement just suggested to remedy these anomalies.

Special arrangements are made for the patients to pay in accordance with their means. One-sixth of the weekly earnings of a patient is the usual charge, but where there are more than three children in a family dependent on their parents, threepence per child for each one above three is deducted from the scale rates.

